# Greater general startle reflex is associated with greater anxiety levels: a correlational study on 111 young women

**DOI:** 10.3389/fnbeh.2015.00010

**Published:** 2015-02-06

**Authors:** Eleonora Poli, Alessandro Angrilli

**Affiliations:** ^1^Department of General Psychology, University of PadovaPadova, Italy; ^2^CNR Neuroscience InstitutePisa, Italy; ^3^CNC – Centro di Neuroscienze Cognitive, University of PadovaPadova, Italy

**Keywords:** anxiety, baseline startle reflex, emotion, EMG, laterality, noise aversiveness

## Abstract

Startle eyeblink reflex is a valid non-invasive tool for studying attention, emotion and psychiatric disorders. In the absence of any experimental manipulation, the general (or baseline) startle reflex shows a high inter-individual variability, which is often considered task-irrelevant and therefore normalized across participants. Unlike the above view, we hypothesized that greater general startle magnitude is related to participants’ higher anxiety level. 111 healthy young women, after completing the State-Trait Anxiety Inventory (STAI), were randomly administered 10 acoustic white noise probes (50 ms, 100 dBA acoustic level) while integrated EMG from left and right *orbicularis oculi* was recorded. Results showed that participants with greater state anxiety levels exhibited larger startle reflex magnitude from the left eye (*r*_109_ = 0.23, *p* < 0.05). Furthermore, individuals who perceived the acoustic probe as more aversive reported the largest anxiety scores (*r*_109_ = 0.28, *p* < 0.05) and had the largest eyeblinks, especially in the left eye (*r*_109_ = 0.34, *p* < 0.001). Results suggest that general startle may represent a valid tool for studying the neural excitability underlying anxiety and emotional dysfunction in neurological and mental disorders.

## Introduction

The startle reflex is a quick and automatic protective response elicited by an abrupt and intense stimulation. It consists of a rapid descending muscular contraction, extending from the head through the trunk and the knees: in humans it can be reliably measured by the extent of a noise-triggered eyeblink. The amplitude of startle response represents an important probe for testing several pharmacological substances and drugs (Longmore et al., 1988; Swerdlow et al., 2000; Duncan et al., 2001; Moberg and Curtin, 2009), for assessing prenatal maturation and neural integrity in newborns (Huggenberger et al., 2011), the affective deficits in neurological patients with amygdala or frontal cortex lesions (Angrilli et al., 1996, 2008), the emotional alteration in psychiatric patients affected by posttraumatic stress disorder (PTSD; Orr et al., 1995; Grillon et al., 1996), panic disorder (Ludewig et al., 2005), bipolar disorder (Giakoumaki et al., 2010) or in psychopathic criminals (Patrick, 1994).

While most of past research has been focused on startle modulation, only little information is available on general startle reactivity (also termed “baseline startle reflex”). The general startle paradigm presents a series of loud acoustic bursts in the absence of any other stimulation or experimental manipulation. It can be considered a measure of the “baseline excitability within the startle reflex circuitry” (Giakoumaki et al., 2010), and is a stable individual neurobiological tract as its heritability is about 70% (Anokhin et al., 2003). In agreement with genetics, amplitude of the general startle reflex showed a good intra-individual coherence across time (Larson et al., 2000), but it also revealed a high inter-individual variability, which has been often considered from researchers as a task-irrelevant noise and therefore subtracted by task-related responses or normalized among participants by *T*-score (equivalent to *z*-score) transformation (Berg and Balaban, 1999; Blumenthal et al., 2005; see for a review of emotion-related startle modulation response in clinical samples: Vaidyanathan et al., 2009). Instead, this variability in general startle reactivity showed a good correlation with the magnitude of threat-potentiated startle (Grillon and Baas, 2002; Bradford et al., 2014) and may reflect the resting state activity of brain areas, including both limbic and paralimbic structures, which have a key role both in the modulation of the startle reflex and in important processes such as sustained fear and anxiety. Startle amplitude is mainly modulated by activation of the nucleus reticularis pontis caudalis which in turn receives projections by several cortical and subcortical regions. At this level, for instances, afferents to the reticular system from both the amygdala and from the orbitofrontal cortex are able to increase arousal and enhance the reflex, and when the above structures are damaged the reflex is inhibited (Angrilli et al., 1996, 2008). Indeed, the amygdala has a fundamental role in fear associated responses to a phasic threat cue but, through its connections to the bed nucleus of the stria terminalis (BNST) it is also involved in sustained threat and to chronic stress response (Davis, 1998). Several studies based on neurological lesions, psychopharmacology and specific anxiety disorders, showed how the absolute startle reflex amplitude might be related to the level of anxiety and the associated physiological activation. Concerning brain lesions, it has been shown that amygdala lesion blocks the potentiation of the startle reflex to fear conditioned cues (Hitchcock and Davis, 1986). In addition, in a patient with a right amygdala lesion, Angrilli et al. (1996) found an overall inhibition of startle reflex magnitude in both eyes, but to greater extent controlaterally, in the left eye. Furthermore, also startle potentiation elicited by fear stimuli was inhibited, a result which points to common shared mechanisms/structures underlying to both general and fear-potentiated startle.

Concerning anxiety disorders, extensive review of the literature with startle probes alone has shown controversial results (Vaidyanathan et al., 2009): some studies found an association between clinical anxiety and an incremented general startle reflex, but these were equivocal and regarded specific subcategories of pathological anxiety. For example, different authors reported an enhanced general startle reflex in war veterans with PTSD (Butler et al., 1990; Orr et al., 1995; Grillon et al., 1996; Morgan et al., 1996; Grillon and Morgan, 1999). Other authors found no general startle differences between PTSD patients and controls (Shalev and Rogel-Fuchs, 1992; Shalev et al., 1992; Metzeger et al., 1999). Ludewig et al. (2005) reported an increased general startle reactivity in unmedicated patients with panic disorder. Kumari et al. (2001) found that patients with obsessive-compulsive disorder had an enhanced startle reactivity and shorter latency as compared to those measured in healthy individuals. Authors explained this result as due to possible abnormalities of corticotrophin-releasing hormone (CRH) in the nucleus reticularis pontis caudalis, which plays a role in the modulation of the general startle reflex amplitude.

The present study aimed to demonstrate, in a large sample of 111 female participants, that part of the large interindividual variance in general startle reactivity is explained by anxiety levels. We chose young subjects because the literature reports that the startle reflex is reduced in elderly participants (Ellwanger et al., 2003; Ludewig et al., 2003). Furthermore, the startle reflex amplitude is different between men and women. For example, two studies demonstrated smaller startle reflex magnitudes in male compared with female participants (Kofler et al., 2001; Bianchin and Angrilli, 2012). In order to make the sample more homogeneous and to avoid gender confounding (males are expected to have lower startles and lower correlations), but also due to greater availability of large female student samples, only women were selected. We hypothesized a positive correlation between state/trait anxiety and the magnitude of the startle reactivity in a general startle paradigm. In line with past literature on dominance of right amygdala in aversive responses (Morris et al., 1999; Baker and Kim, 2004) and of left EMG facial responses (Schwartz et al., 1979; Dimberg and Petterson, 2000) controlled by right hemisphere, we expected that the left startle, under the control of the right amygdala (Angrilli et al., 1996), would be correlated with anxiety levels.

## Methods

### Participants

Participants were 111 female students in introductory psychology classes at the University of Padova, who participated for course credit. Mean age was of 23.32 years (SD = 2.13). Because startle reflex magnitude is influenced by smoke, caffeine and alcohol (Swerdlow et al., 2000; Duncan et al., 2001; Moberg and Curtin, 2009), all participants were asked to refrain from smoking cigarettes and drinking coffee or alcohol 2 h before the beginning of the experimental session. The procedure was approved by the local Psychology Ethics Committee.

### Material and procedure

After they signed the informed consent form, participants completed the Trait form (Y2) of the State-Trait Anxiety Inventory (STAI; Spielberger, 1983). The inventory is typically divided into two sections of 20 items each: the State form (Y1) assessing how participants feel in that particular moment, the Trait form (Y2) estimating how they generally feel.

The procedure was carried out by following current guidelines on EMG and startle reflex recordings (Fridlund and Cacioppo, 1986; Blumenthal et al., 2005). To control for the confound of hearing problems on startle response (the number of young people suffering from hearing loss is increasing due to the frequent use of headphones and loud volumes) individual’s hearing threshold was assessed with an on-line test that measured the relative sensibility of the ears at different frequencies.^1^ A Sennheiser HD-202 closed headphone was used. All participants reported normal hearing capacity (threshold level: below or equal 20 dB).

Participants were then seated in a recliner in a small, dimly lit room and electrodes for blink reflex registration were placed. The acoustic startle stimulus consisted of a 50 ms, 100 dB(A) burst of white noise with instantaneous rise time, presented binaurally through stereophonic headphones. A total of 10 unsignaled acoustic startle probes were delivered with an inter-trial interval varying between 9 and 23 s. Participants were simply instructed to watch a fixation point and ignore the brief noises heard over the headphones. After this procedure, participants were asked to rate the aversiveness of the noise on a 0 (“not aversive”) to 10 (“extremely aversive”) Likert Scale.

The State form (Y1) of the Anxiety Inventory was administered at the end of the experimental session, before debriefing the participant.

### Physiological recording

A LabVIEW program (Angrilli, 1995) was used for data acquisition and analysis. In line with current guidelines (Blumenthal et al., 2005), the eyeblink component of the startle reflex was measured from the *orbicularis oculi* muscle with 6 mm Ag/AgCl cup electrodes placed below the participant’s left and right eyes. The ground electrode was placed on the forehead and a conductive gel was used to improve electrical contact: all electrodes were checked for an impedance below 5 Kohm. The raw EMG signals were amplified with a gain of 10000, and filtered with a 16 Hz first order high-pass and a 340 Hz second order low-pass. The signals were rectified and integrated with a 100 ms time constant integrator. Rectified and integrated EMG data were sampled at a rate of 250 Hz. Raw signals were visually inspected in order to reject rare artifacts. The valid trials were then averaged and the latency of each participant’s peak was used to identify a 20 ms window centered on the peak. Startle reflex magnitude was scored as the mean value of the integrated EMG signal in the 20 ms peak-centered time-window for both the left and the right eyes (Angrilli et al., 1996, 2008).

### Data analysis

State and Trait Anxiety raw scores collected by the STAI (Spielberger, 1983) were used for data analysis. The association between blink reflex magnitude, measured in the left and right eyes, and anxiety, was analyzed using Pearson’s correlation with critical *p* = 0.05. In addition, also the correlations between subjective noise aversiveness, blink magnitude and anxiety levels were computed.

## Results

No associations were found between startle reflex magnitude and Trait anxiety score measured at the beginning of the experimental session (*r*_109_ = 0.18, and *r*_109_ = 0.12, ns. left and right eye, respectively).

**Figure 1:**
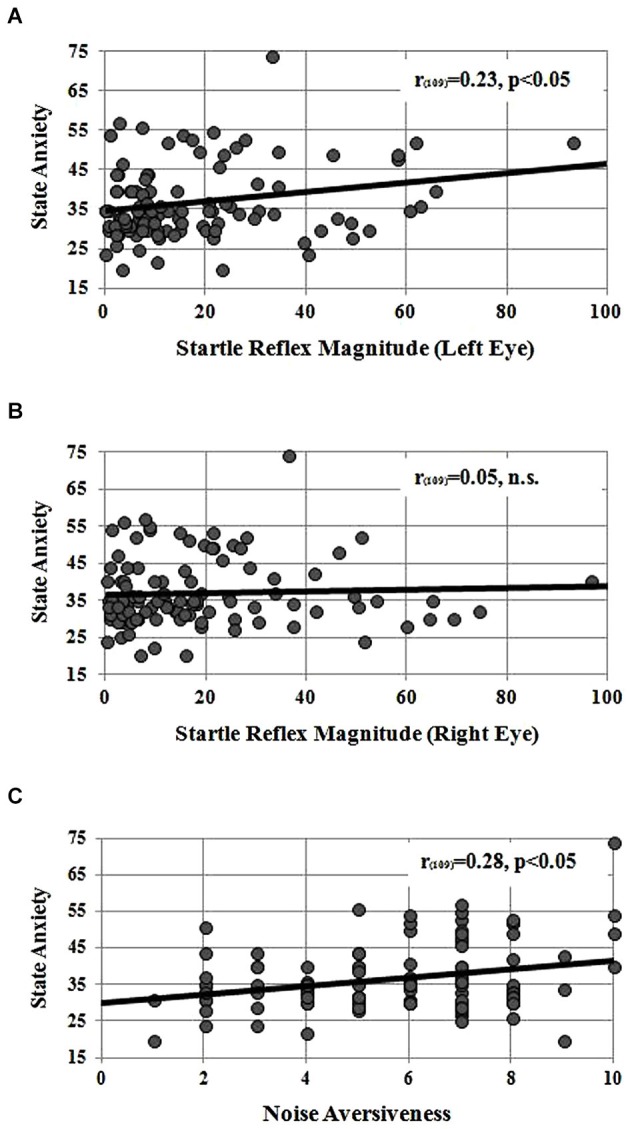
**Panel (A) correlation between startle reflex magnitude, measured in microvolts below the left eye, and the State Anxiety raw score measured at the end of the experimental session**. Panel **(B)** correlation between startle reflex magnitude measured from the right eye and the State Anxiety raw score. Panel **(C)** correlation between state anxiety raw score and the subjective noise aversiveness measured on a 0–10 analog scale.

Instead, left startle reflex magnitude significantly correlated with the State anxiety measured at the end of the session (*r*_109_ = 0.23, *p* < 0.05; see Figure 1A): the higher the blink magnitude, the higher the State Anxiety score. No significant correlation was found between startle reflex magnitude of the right eye and State anxiety measured at the end of the session (*r*_109_ = 0.05, ns, see Figure 1B). Figure 1C displays the correlation found between State anxiety and subjective noise aversiveness rated on a 0–10 Likert scale. A significant positive association revealed that participants with greater State anxiety also reported greater perceived aversiveness of the acoustic noise heard through the headphones (*r*_109_ = 0.28, *p* < 0.05).

**Figure 2:**
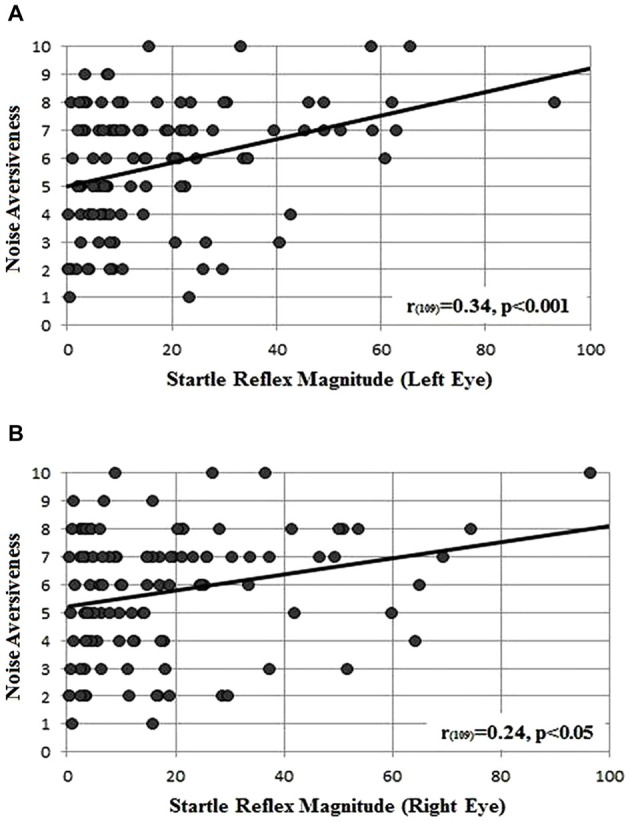
**Correlation between the startle reflex magnitude measured in microvolts, below the left (A) and (B) right eye and the perceived noise aversiveness (scale 0–10)**.

As shown in Figure 2, both the left (Figure 2A) and right (Figure 2B) startle reflex amplitudes showed a significant positive correlation with the perceived noise aversiveness (*r*_109_ = 0.34, *p* < 0.001 and *r*_109_ = 0.24, *p* < 0.05, respectively): the higher the startle reflex magnitude, the higher the subjectively perceived aversiveness of the acoustic burst of noise.

## Discussion

With the present experiment we aimed to demonstrate a relationship between anxiety levels and general startle amplitude. To date, most of past research on healthy participants has been focused on startle modulation rather than on general startle, as the latter was considered relatively independent from the task and irrelevant with respect to its modulation. For this reason, in most studies general startle has been used to normalize (Berg and Balaban, 1999), through scaling, the task-dependent startle modulation, thus canceling out most of individual differences in this measure. This approach has underestimated and often masked individual differences in general amplitude of the reflex: for instance, while review on affect-modulated startle did not report gender differences in fear-potentiated startle (Grillon and Baas, 2003; Vaidyanathan et al., 2009), only a few studies which did not normalize startle responses, revealed a clear gender effect in general startle amplitude (Kofler et al., 2001; Bianchin and Angrilli, 2012). However, as shown by a recent review (Vaidyanathan et al., 2009), clinical research on samples of patients with anxiety disorders and PTSD (Butler et al., 1990; Orr et al., 1995; Grillon et al., 1996; Morgan et al., 1996; Grillon and Morgan, 1999) evidenced that general startle can be enhanced in these individuals, although this effect has not been consistently demonstrated in all PTSD studies, possibly for the large variety of the methods, samples and context. One methodological issue which may explain inconsistencies across clinical studies using general startle is represented by contextual fear elicited by instructions and tasks: when participants sign informed consent that a threat session will occur during experiment, this might increase in an unpredictable and uncontrolled way fear and general startle response in all participants, or in part of them. In the present experiment the startle probe alone session was not followed or associated to any threat condition/session. Furthermore, given the limited number of human studies on general startle, probably due also to the weak and inconsistent association of this probe with psychological factors in general, we expected to find a small but significant correlation between general startle and anxiety levels. Therefore, to detect this small effect, we sought to enroll a relatively large sample of students (above one hundred) in the study. Results revealed a significant positive correlation between left startle amplitude and state anxiety measured during the experimental session, this effect was substantial and significant only for the left startle, the correlation of anxiety with reflex amplitude measured from the right eye was negligible. A past study provided the first hint of a possible hemispheric asymmetry in acoustic startle potentiation circuit (Bradley et al., 1996), a result which was based on the monoaural startle probe paradigm and which can not be directly compared with our binaural probe condition. Furthermore, the effect from the left startle was expected because it was in line with past literature showing the relevance of the right amygdala-left startle in fear emotional control (Angrilli et al., 1996; Morris et al., 1999; Baker and Kim, 2004), but also with literature showing the left dominance of EMG facial expressions (Schwartz et al., 1979; Dimberg and Petterson, 2000). Interestingly, a recent study investigated the correlation between anxiety and startle amplitude and habituation from the right eye (Campbell et al., 2014) using a different psychometric tool, the ASI 3 (Anxiety Sensitivity Index), more sensitive to specific fears than to anxiety itself, and correlations were significant only for startle habituation (a new interesting parameter), not for startle amplitude, a result consistent with our experiment because startle amplitude from the right eye was not correlated with anxiety score.

It is worth to note that past research has highlighted a relation between general startle and startle potentiation: subjects with the highest absolute startle had also the largest threat-related startle potentiation (Grillon and Baas, 2002; Bradford et al., 2014). This relationship suggests that the same structures, mechanisms and processes (fear, anxiety) involved in startle potentiation are also involved in general startle. In line with this, absolute startle amplitude is controlled by several regions (amygdala, prefrontal cortex, reticular formation; see Angrilli et al., 1996, 2008) which in good part control also the fear-potentiated startle (Davis, 2006).

In the present study only state anxiety was correlated with startle amplitude, probably because in laboratory setting, trait anxiety, which is a stable personality factor, tracks less precisely current (state) anxiety induced by the novel laboratory context. The latter may be sufficient to increase, in the most sensitive healthy subjects, contextual anxiety and apprehension (i.e., increased state anxiety) which in turn leads to heightened general startle reflex.^2^ This observation is also supported by past experiments in which general startle was strongly correlated with startle potentiation with uncertain vs. certain threat (Bradford et al., 2014). Increased anticipation of uncertain threat is one leading and common feature of anxiety disorders and startle reflex has shown to be increased in ecological virtual environments with unpredictable contexts (Grillon, 2008). An additional interesting and novel result is the significant positive correlation found between perceived noise aversiveness and startle amplitude. Those individuals who exhibited the largest startle reported also a greater subjective noise annoyance, this was evident especially for the left startle and, to a less extent yet still significant, for the right startle: this correlation again points on the greater sensitivity of left startle reflex to danger-related noise. The correlation with perceived aversiveness can be explained by the observation that in the afferent regions of amygdala there are neurons sensitive to both strong intensity of sensory stimulation (Armony and LeDoux, 1997; Yeomans et al., 2002) and to rapid change in stimulus intensity (a shorter rise time is associated with larger startle amplitude, see Turpin et al., 1999), a condition which in nature often marks dangerous situations. From another domain, high intensity stimuli modulate arousal and the reticular formation activation which in turn plays a role in modulating the general startle reflex as shown in past studies with neurological patients reporting a dampened arousal and startle (Angrilli et al., 1996, 1999). Coherently, the significant correlation between state anxiety and perceived noise aversiveness confirms that, at cortical subjective level, individuals with a triggered large reflex are also the most scared and annoyed by the high acoustic loudness of the stimuli. Conversely, patients with lesion in the OFC evidenced an inhibited general startle amplitude together with a significant reduced perception of aversiveness of the acoustic probe (Angrilli et al., 2008).

The three variables which have been correlated, startle amplitude, state anxiety and perceived aversiveness evidenced a consistent relationship among them. However, it must be highlighted that, overall, the correlations were significant but did correspond to small effect sizes (between 0.2 and 0.3) and to small shared variances, as two very different and complex domains, each one influenced by a large number of variables, have been put in relation: general startle reflex which is an implicit physiological measure controlled and modulated by a number of cerebral structures, processes and stimuli, and state anxiety, an explicit verbal self-evaluation measure which is biased by many situational variables and for which participants might have only a partial insight and awareness. Indeed, not all individuals are able to rate and satisfactorily describe their level of apprehension and anxiety, as this state as well as precise introspection into it can not be comprehensively described by a verbal report. All these aspects make the two different domains measured in the present investigation only partially associated. It is important to highlight that, in psychophysiological research, e.g., in emotion field, the concordance among different domains, e.g., the explicit verbal domain (together with participant’s awareness of her internal state) with the implicit physiological measure (e.g., skin conductance, heart rate, evoked potentials, etc.), is a debated and problematic issue (Hollenstein and Lanteigne, 2014). In light of this, results of the present study are important because a relationship was found between state anxiety and left startle reflex in healthy individuals at rest, suggesting that the general blink response, so far considered a spurious variable which must be canceled out by normalization procedures, in future investigations could unmask important relationships with other individual differences and with mental and personality disorders such as schizophrenia and bipolar disorder (Thaker, 2008), schizotypical (Light and Braff, 2003), borderline (Ebner-Priemer et al., 2005) and psychopathic disorders (Patrick, 1994). Till now these illnesses have been mostly investigated with pre-pulse inhibition or startle potentiation paradigms, but in clinical research general startle may become an interesting additional and simple tool for estimating anxiety and emotion-related neural excitation in the above mentioned mental disorders.

## Author contributions

Alessandro Angrilli conceived and designed the experiment, Eleonora Poli collected and analyzed all physiological and behavioral data, Alessandro Angrilli and Eleonora Poli wrote and edited the manuscript.

## Conflict of interest statement

The authors declare that the research was conducted in the absence of any commercial or financial relationships that could be construed as a potential conflict of interest.

## Footnotes

^1^http://www.phys.unsw.edu.au/jw/hearing.html

^2^To be noted that contextual apprehension naturally induced by novel environment represented by the laboratory is a weak natural condition with respect to the cued fear-related instruction provided in experiments including threat, the latter condition may lead to a ceiling effect in general startle reflex which reduces variances and correlations, an issue which deserves further research.
